# Continuous glucose monitoring for children with hypoglycaemia: Evidence in 2023

**DOI:** 10.3389/fendo.2023.1116864

**Published:** 2023-01-23

**Authors:** Chris Worth, Lucy Hoskyns, Maria Salomon-Estebanez, Paul W. Nutter, Simon Harper, Terry G.J Derks, Kathy Beardsall, Indraneel Banerjee

**Affiliations:** ^1^ Department of Paediatric Endocrinology, Royal Manchester Children’s Hospital, Manchester, United Kingdom; ^2^ Department of Computer Science, University of Manchester, Manchester, United Kingdom; ^3^ Section of Metabolic Diseases, Beatrix Children’s Hospital, University of Groningen, Groningen, Netherlands; ^4^ Department of Paediatrics, University of Cambridge, Cambridge, United Kingdom; ^5^ Faculty of Biology, Medicine and Health, University of Manchester, Manchester, United Kingdom

**Keywords:** hypoglycaemia, continuous glucose monitoring, children, hyperinsulinism, glycogen storage disease, prematurity

## Abstract

In 2023, childhood hypoglycaemia remains a major public health problem and significant risk factor for consequent adverse neurodevelopment. Irrespective of the underlying cause, key elements of clinical management include the detection, prediction and prevention of episodes of hypoglycaemia. These tasks are increasingly served by Continuous Glucose Monitoring (CGM) devices that measure subcutaneous glucose at near-continuous frequency. While the use of CGM in type 1 diabetes is well established, the evidence for widespread use in rare hypoglycaemia disorders is less than convincing. However, in the few years since our last review there have been multiple developments and increased user feedback, requiring a review of clinical application. Despite advances in device technology, point accuracy of CGM remains low for children with non-diabetes hypoglycaemia. Simple provision of CGM devices has not replicated the efficacy seen in those with diabetes and is yet to show benefit. Machine learning techniques for hypoglycaemia prevention have so far failed to demonstrate sufficient prediction accuracy for real world use even in those with diabetes. Furthermore, access to CGM globally is restricted by costs kept high by the commercially-driven speed of technical innovation. Nonetheless, the ability of CGM to digitally phenotype disease groups has led to a better understanding of natural history of disease, facilitated diagnoses and informed changes in clinical management. Large CGM datasets have prompted re-evaluation of hypoglycaemia incidence and facilitated improved trial design. Importantly, an individualised approach and focus on the behavioural determinants of hypoglycaemia has led to real world reduction in hypoglycaemia. In this state of the art review, we critically analyse the updated evidence for use of CGM in non-diabetic childhood hypoglycaemia disorders since 2020 and provide suggestions for qualified use.

## 1 Introduction

In 2023, non-diabetes hypoglycaemia remains a major global problem for children. Its effects are far reaching, with impacts on quality of life (1, 2), health economics (3), hypoglycaemia fear (4), reaching beyond the individual to the extended family (5, 6). Although recent studies (7), complimenting previous work (8, 9), have suggested a lesser effect of transient neonatal hypoglycaemia (10), there remains little doubt of the impact of severe childhood hypoglycaemia on neurodevelopmental delay, particularly in those children with severe and recurrent hypoglycaemia due to congenital hyperinsulinism (CHI) (9–11).

Essential to all hypoglycaemia management, irrespective of the cause, is the detection, prediction and prevention of episodes through glucose testing (12, 13). The first of these three tasks has been traditionally performed by fingerprick blood glucose testing (13), with prediction and prevention reliant on clinical skill and patient experience. However, over recent years, all three tasks are increasingly being performed by continuous glucose monitoring (CGM) in either its raw form or through its manipulation by modern computer algorithmics. For people living with diabetes, CGM and associated predictive algorithms are widely used and well established in the reduction of hypoglycaemia (14–17) and cost-effectiveness (18–20). However, for those with a non-diabetes hypoglycaemia disorder, the utility in diabetes has not been replicated and CGM has not been established in routine clinical practice.

The use of CGM in rare hypoglycaemia disorders is a rapidly evolving and expanding field. In this review we have followed on from a comprehensive review in 2020 (13), to provide an update on improvements in the technology and utility of CGM focusing mainly on CHI, glycogen storage diseases (GSD) and neonatal prematurity. We reflect on our predictions from 2020, synthesise current understanding and look to the future.

## 2 Accuracy

We have detailed the background to accuracy assessments in CGM elsewhere (13) but it is worth outlining the two differing approaches to accuracy assessment: 1) pairing CGM values with fingerprick glucometer values and measuring difference; 2) evaluating the ability of CGM devices to ‘detect’ hypo(or hyper)glycaemia within a time window and thus utilising to a fuller extent the semi-continuous nature of CGM. Measures of accuracy differ widely throughout the literature, but the former is more commonly used and tends to incorporate mean absolute relative difference (MARD), mean absolute difference and hypoglycaemia sensitivity/specificity. A summary of CGM accuracy studies by various groups using different CGM devices in non-diabetes hypoglycaemia is presented in **Table 1**.

**Table 1:** Accuracy data for CGM use in non-diabetes childhood hypoglycaemia disorders.

Publication	Patient group	Device	MARD (%)	MD (mmol/L)	MAD (mmol/L)	R^2^	Hypo sensitivity
*Beardsall ‘05(21)*	Neonates	Medtronic MiniMed	—	-0.1	—	0.87	N/A
*Beardsall ‘13(22)*	Neonates	Medtronic System Gold	—	—	—	0.94	(2.6mmol/L) 17%
*Win(23)*	Neonates (+/- CHI)	Medtronic Paradigm OR Dexcom G4	11.0	—	—	—	(2.6mmol/L) 59%
*Vijayanand(24)*	Neonates (+/- CHI)	Dexcom G4	13.1	+0.3	—	—	(3.5mmol/L) 78% (3.0mmol/L) 54%
*Alsaffar(25)*	CHI	Abbott Freestyle Libre	17.9	+0.3	—	0.70	(3.5mmol/L) 52%
*Rayannavar(26)*	CHI	Dexcom G5	17.5	-0.4	—	—	(3.9mmol/L) 86% (3.0mmol/L) 66%
*Worth(27)*	CHI	Dexcom G6	19.3	+0.4	0.9	—	(3.9mmol/L) 52% (3.5mmol/L) 45% (3.0mmol/L) 40%
*Kasapkara(28)*	GSD	Medtronic	—	—	—	0.74	—
*Herbert*(29)	GSD	Dexcom G4	—	—	—	0.57	—
*Rossi*(30)	GSD	Dexcom G6	—	+0.9	—	—	—

MARD, mean absolute relative difference; MD, mean difference; MAD, mean absolute difference; R^2^, correlation coefficient between blood glucose and CGM glucose levels; Hypo, hypoglycaemia.

### 2.1 Neonates

Beardsall et al. first evaluated the accuracy of CGM devices in neonates in 2005 (21) and later in 2013 (22); they reported a correlation coefficient of 0.69-0.94 with safe results on an error grid (albeit one designed for those with diabetes). However, hypoglycaemia sensitivity was found to be only 17%. More recent results from the same group showed a relatively small MARD of 11% but a hypoglycaemia sensitivity of only 59% with the latest devices and technologies (23). These calculations were based on a lower threshold for hypoglycaemia (<2.6mmol/l) than is usually used outside the neonatal unit. Furthermore, as described above, sensitivity is based on point comparisons of accuracy which can underestimate the clinical value of sensor glucose trends in detecting hypoglycaemic events. Recent work in Australia by Vijayanand et al. (24) has confirmed the poor hypoglycaemia sensitivity seen in this group with results of 54% when using point comparisons.

### 2.2 Childhood hypoglycaemia disorders

CGM is not routinely used in patients with CHI and therefore data is relatively sparse (**Table 1**). In the first evaluation of CGM in CHI, Alsaffar et al. (25) reported a hypoglycaemia (3.5mmol/L) sensitivity of only 52% but did not report a MARD. While an evaluation of a more up to date device by Rayannavar et al. (26) showed a better hypoglycaemia sensitivity of 86%, this was calculated using a higher cut-off for hypoglycaemia (3.9mmol/L), as is standard practice in some countries. When hypoglycaemia <3.0mmol/L was investigated, a low sensitivity of 66% was demonstrated. As existing error grids (such as Parks and Clarke) are designed for evaluation of CGM accuracy for those with diabetes, they have not been used as standard in assessments in CHI. Recently Worth et al. (27) developed an expert-consensus error grid for use in CHI and used this to evaluate the accuracy of one of the most recent CGM sensors, the Dexcom G6. Results suggested the presence of significant clinical risk in the use of CGM for patients with CHI due to poor device accuracy on error grid analysis and hypoglycaemia sensitivity of only 45%. Analysis of the ability of the Dexcom G6 to detect glucometer-measured hypoglycaemia within a 30 minute window was marginally better but still unreliable at 51% (27).

Equally, CGM is also not used routinely in patients with GSD and assessments of CGM accuracy for this group have been largely incomplete (**Table 1**). These demonstrate correlation between CGM and glucometer values but the magnitude of error has not been reported. Papers (28, 29) report mean difference or correlation but due to the presence of both overestimation and underestimation, and no report of mean *absolute* difference, it is impossible to determine the average magnitude of errors. Rossi et al. (30) went on to evaluate CGM error by glucose value and also between those with GSD1a and healthy volunteers. They found that CGM overestimation was worse for those with GSD1a and at glucose values <3.9mmol/L, thereby increasing the risk of missed hypoglycaemia for the most vulnerable groups at the time of greatest need.

## 3 Efficacy of CGM to detect and prevent hypoglycaemia

We have previously summarised the efficacy of CGM for children with non-diabetes hypoglycaemia due to various conditions (13). Here we summarise recent developments in the field with regards to the conventional use of CGM to detect and prevent hypoglycaemia by simple provision to patients and clinicians. The non-conventional use of CGM is discussed later in Section 7.

### 3.1 Neonates

Previously summarised studies (13) have demonstrated the utility of CGM to reduce painful procedures, detect unsuspected hypoglycaemia and reduce *hyper*glycaemia. More recently, Fernández Martínez et al. (31) confirmed the ability of CGM to detect unsuspected and prolonged hypoglycaemia in very low birth weight (VLBW) neonates. Win et al. (23) have since demonstrated significant fluctuations in glucose in neonates; more pronounced in those with CHI. The same group recently published the results of an international, multi-centre RCT investigating the use of CGM in preterm neonates and clearly demonstrated a reduction in hypoglycaemia and hyperglycaemia for those in the CGM group (32) encouraging CGM as a potential tool for regular use in the neonatal intensive care unit.

### 3.2 Hypoglycaemia associated with rare endocrine conditions

At the time of our previous review in 2020, there was no evidence for CGM reducing hypoglycaemia for children with any endocrine conditions other than diabetes mellitus. In the absence of larger scale studies, we discussed (13) minimal evidence for use of CGM for both adults and children with adrenal insufficiency (AI) and the anecdotal reports of CGM use for those with CHI.

Further single-case, anecdotal reports of utility of CGM in CHI (33) and hypopituitarism (34) have since been published. Importantly however, Worth et al. have recently published non-randomised data on CHI patients with periods of blinded and unblinded CGM (35); suggesting that the simple provision of CGM (without expert or algorithmic interpretative support) does *not* reduce hypoglycaemia for those with CHI. The addition of interpretative algorithmic or clinical support is discussed in Section 7. However, at the time of writing, there are no comprehensive studies evaluating the efficacy of CGM to reduce hypoglycaemia for children with endocrine hypoglycaemia.

### 3.3 Hypoglycaemia associated with rare hereditary metabolic disorders

We have previously outlined (13) the utility of CGM to detect unsuspected hypoglycaemia and facilitate manipulation of diet and treatment for patients with GSD. Previous anecdotal reports highlighted the utility of retrospective CGM data analysis but advised against the provision of real-time CGM to patients for fear of inappropriate treatment alterations (36). Since our previous review, there have been further anecdotal reports of CGM utility in the detection of glycaemic variability and excursions for patients with metabolic causes of hypoglycaemia (37–39) but no systematic evaluations of the use of CGM to actually prevent or reduce hypoglycaemia.

## 4 Family perspectives

Our previous review discussed families with CHI and GSD reporting marginal benefit from the use of CGM as secondary outcomes of studies. Anecdotally, families found glucose trends helpful. Since 2020, the significant increase in the use of CGM in hypoglycaemia disorders has led to an increase in literature regarding families’ perceptions of this emerging technology, described below.

### 4.1 Patient charity reports

Patient charities fulfil a vital role of providing support to those with hypoglycaemia conditions but also provide an important window into the views and opinions of families. In a recent unpublished study (summarised in an opinion paper (40)), the UK Children’s Hyperinsulinism Charity (UK CHC) reported that families with CHI find CGM: offers a safety net, improves quality of life, and reduces worry. Patients reported (40) difficulty in access to CGM and a call was made for wider availability for families with CHI. While this survey is likely subject to significant positive sampling bias, it does offer an important insight into the opinions of some families with CHI.

The charity Congenital Hyperinsulinism International (CHI) recently revealed that 45.7% of respondents to a global registry use CGM but that access to devices is often a problem and trust in the data generated is often low (2). They also report that families generally find devices useful but that patients experience problems with poor accuracy (6). Again, this is likely open to sampling bias but offers an important user-perspective. Within GSD, CGM is a much higher research priority for healthcare professionals than it is for patients and carers who rank it as a lower priority (41).

### 4.2 Qualitative studies

While patient organisations have called for wider access to CGM, it is important to formally assess families’ experiences of CGM to actively seek out both positive and negative views. As recently highlighted by Peeks et al. (42), “glucose management as assessed with CGM should be balanced against psychosocial well-being and quality of life” which cannot be assumed to be higher with CGM than without.

In CHI patients, Auckburally et al. (43) undertook semi-structured interviews with families who had been provided with a CGM for 12 weeks as part of a research project. As there was no existing information on CHI families’ experiences of CGM, the authors performed a thematic analysis to identify themes important to patients and their families. Such detailed analysis revealed a rich and complex mixture of attitudes towards CGM. Families reported positive feelings about CGM’s function as an educational tool which could motivate behavioural changes to prevent hypoglycaemia. However, the problematic issues of poor accuracy and irritating alarms were raised by all participants.

In order to better understand the reasons for a high rate of dissatisfaction with CGM seen in CHI families, Ahmad et al. (44) performed semi-structured telephone interviews with those who had discontinued use. Primary reasons for discontinuation were pain, device inaccuracy, issues with technical setup and 90% of those surveyed thought that CGM device use would have been easier if their child had been a different age (either younger or older) (44). Comprehensive assessments of families’ experiences of CGM, with a focus on the reduction of selection bias, are essential in the journey to establish CGM as a therapeutic option for paediatric hypoglycaemia disorders. The authors are aware of two separate studies aiming to achieve this for families with CHI and the results are eagerly awaited.

## 5 Barriers to the use of CGM

In our 2020 review we highlighted the barriers to wider use of CGM in paediatric hypoglycaemia disorders and to date there are no improvements with regards to lag time, alarms or fingerprick testing. However, with regards to clinician inertia and cost, an update is worthwhile.

### 5.1 Clinician inertia and usability

Over the last three years, the authors have noticed a significant increase in the interest in CGM by clinicians working in paediatric hypoglycaemia disorders. There is now less suspicion of the technology and a higher acceptance of using CGM as a routine part of care. This is mirrored in the significant increase in publications relating to CGM in both hypoglycaemia disorders and neonatology. However, the interest and marketing strategy of device manufacturers remains firmly focused on diabetes mellitus, precluding wider adoption and development specific to hypoglycaemia.

### 5.2 Cost and widening access

As CGM technology develops, it is important that the availability of devices is considered, especially for those in low-income countries (LICs) and for patients with rare diseases. These groups are often marginalised and disadvantaged in the commercially-driven push for technological progression but efforts must be made to minimise access inequalities (45). As a technology, CGM could arguably have significant impact in LICs due to the added burden of hypoglycaemia from malaria, malnutrition, diarrhoea and sepsis (46). Additionally, for people living with diabetes, access to insulin is often intermittent in LICs (47), leading to hypoglycaemia and hyperglycaemia. CGM would also be highly valuable in the neonatal setting as capacity for regular glucose monitoring in neonatal units in LICs is often limited and neonatal mortality is high (48). Indeed, neonatal hypoglycaemia is often present in otherwise uncomplicated newborn infants, and recognition and treatment may have a significant impact on neonatal outcomes (49, 50).

Moreover, the long-term impacts associated with childhood hypoglycaemia, such as neurodisability, epilepsy and reduced cognitive function (9, 51) have a higher burden in LICs, being poorly understood by wider society and suboptimally managed due to meagre resources (52–54). So, while the costs of CGM may be high, its implementation may enable faster, accurate treatment modification, improving outcomes (38) and likely contributing to value based healthcare in both common, high volume disease (55) and rare, low volume disease such as GSD (56). However, it is important to recognise that technology developed for a high-income setting is not always appropriate for LICs where the environment is different; there can be extremes of temperatures, intermittent access to internet and electricity, high levels of dust and minimal access to engineers to repair devices (50, 57–59). A target product profile (TPP)-based approach has been developed to identify key specifications for product innovation in LICs. This approach has been particularly successful in development of neonatal devices, most notably in bubble CPAP, and a similar approach should be considered in the development of CGM devices (50, 60).

## 6 Updates on previously suggested developments

In our 2020 review we predicted that future developments would be focused on CGM device technology and predictive hypoglycaemia algorithms. Here we provide an update on the developments in these areas over the last three years before moving on to discuss alternative and novel areas for CGM use in Section 7.

### 6.1 CGM device technology

The direction for CGM device technology development continues towards miniaturisation, with a focus on reducing the invasive nature of some CGM devices. Dexcom^®^ have since released the G7 device which is smaller, thinner and predicted to be more accurate. Abbott^®^ have released the Freestyle Libre 3, also smaller and thinner and now offering real time readings with optional alerts. Eversense^®^ now have an implantable sensor with a six month wear time and requiring only a single calibration per day.

There has also been significant interest in the last few years on optical sensors that detect photons to determine the glucose concentration via the interaction between glucose molecules and different wavelengths of light (61). Other sensor developments focus on the non-invasive measurement of sweat, urine, saliva, tears (62) and even thermal monitoring (63); however, these ideas have not yet translated to a commercially viable stage.

### 6.2 Predictive hypoglycaemia algorithms

**Figure 1:**
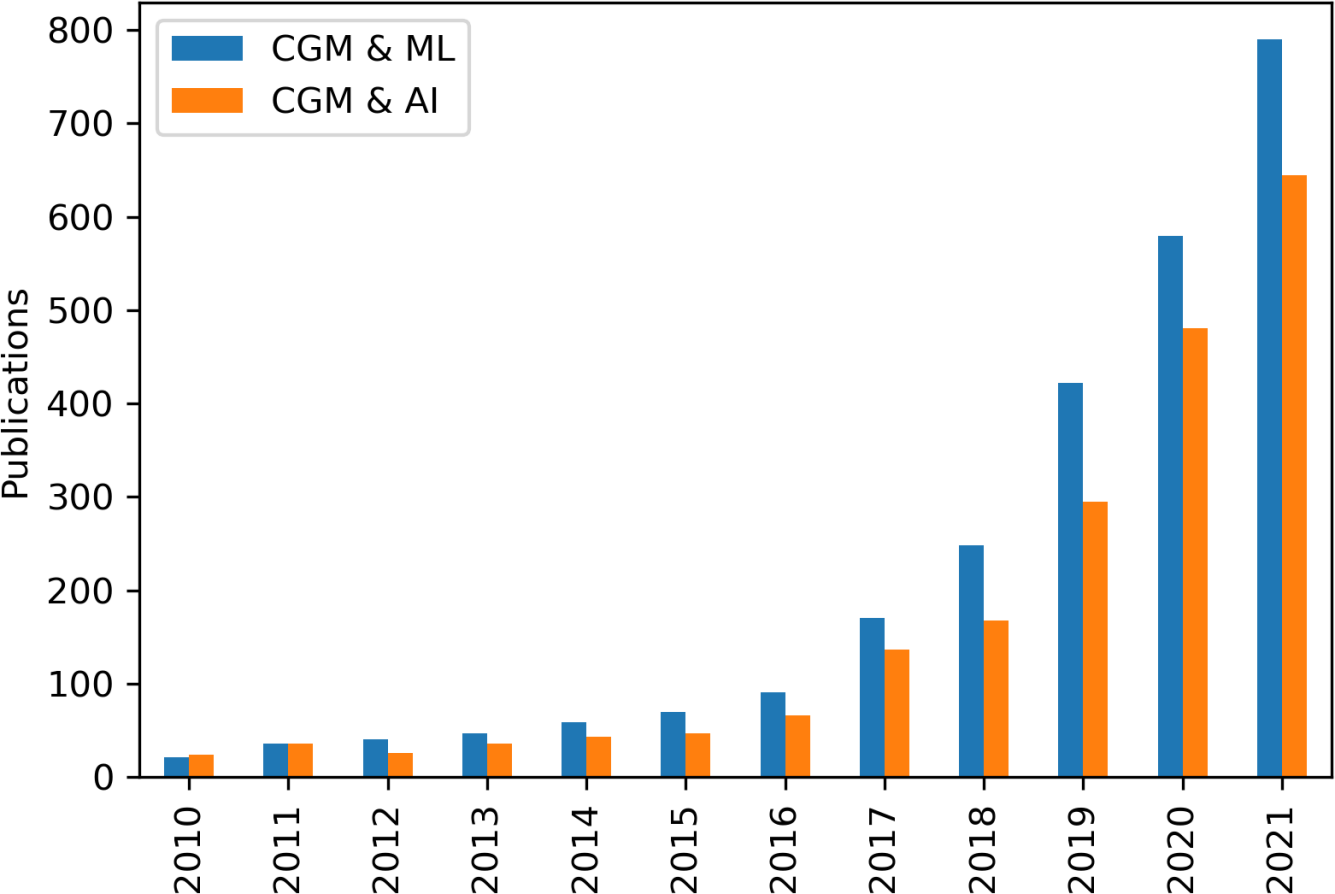
Publications by year with the search terms "continuous glucose monitoring" (CGM) and "machine learning" (ML) or “artificial intelligence” (AI), found on Google Scholar.

Our 2020 review (13) outlined the background to the use of predictive algorithms for hypoglycaemia and the different forms that these can take; physiological, data-driven, and hybrid (64). While non-machine learning algorithms such as Model Predictive Control have been beneficial for adults (65) and neonates (66) using closed loop insulin delivery, these systems are of no use to the majority of patients with rare hypoglycaemia disorders whose hypoglycaemia is not caused by exogenous insulin. Work in the field of data-driven predictions continues to expand rapidly in diabetes and artificial intelligence and machine learning methods using large historical datasets continue to be used to derive theoretical prediction models (**Figure 1**). While, multiple groups have continued to publish increasingly accurate in-silico algorithms (67–70), these have been evaluated by systematic review (71) and meta-analysis (72) and found to have insufficient ability to detect and prevent hypoglycaemia. The authors conclude that improvement is required before application in clinical settings. As suggested, these algorithms have been evaluated in-silico only with no conclusive examples of Machine Learning-driven predictive algorithms reducing hypoglycaemia in the real world.

Decision Support Systems (DSS) are an extension of glucose predictive algorithms and facilitate decision making (e.g. food intake) based on various inputs (e.g. CGM data) and predicted outcomes (e.g. hypoglycaemia). Recent DSSs have shown in-silico (73) and possibly real world (74) reduction in hypoglycaemia through modification of insulin dosing for people living with diabetes. However, Tyler et al. (75) note in their systematic review that “it has not yet been shown that a DSS can improve time in range in human studies” and more work is required. Vitally, all DSSs focus on the use of exogenous insulin as either an input or output and are therefore of no use to those with a rare hypoglycaemia disorder such as CHI or GSD but may have potential in neonates on insulin therapy (66).

## 7 Novel directions and a possible future for CGM in hypoglycaemia

So far we have provided updates on areas covered in our previous review. In this section we move on to discuss novel areas and uses for CGM which have either emerged since 2020 or are now gaining prominence. Person-centred outcome measures have been defined for type 1 diabetes (76, 77) but are currently lacking for rare hypoglycaemia disorders. This causes difficulty in comparing studies and evaluating day to day impact for patients. Consensus, person-centred outcomes would greatly enhance routine healthcare and research for these groups, particularly with regards to emerging but as yet unproven technologies such as CGM.

### 7.1 CGM to elicit patterns and digital phenotypes

There is increasing recognition of phenotypes beyond those classically described by physical traits or cellular changes. Most recently established is the “digital phenotype” (78). The digital phenotype covers both aspects of behaviours related to technology such as social media use use as well as behaviours measured by technology such as heart rate monitors, accelerometers and CGM. These new measures facilitate a more comprehensive and individualised picture of patients’ health and contribute to “P4 medicine” (79); allowing for a predictive, preventative, personalised and participatory approach to management.

Worth et al. (80) took the first steps towards extending the digital phenotype of CHI with their analysis of retrospectively collected CGM data. Previously collected CGM data was used to identify periods of high hypoglycaemia risk in the early morning in patients with CHI; opening the door for targeted interventions on a group and individual level. Further work by this group (81) investigated patterns of hypoglycaemia at an individual level and found that each patient with CHI had clear and individual *weekly* patterns for repeated hypoglycaemia. Peeks et al. (42) performed a similar analysis in patients with hepatic GSD to provide the first insight into CGM profiles in this patient group and similarly concluded that analysis on a group level was of some use but improved when performed on an individual basis.

Further contributions to the digital phenotypes of hypoglycaemia disorders have been made by Rossi et al. (30) who provided CGM metrics for glycaemic variation and control in adult patients with GSD1a and compared this to healthy volunteers. Worth et al. (82) performed a similar analysis for patients with CHI on a larger scale (3.4 million data points) but without healthy controls to establish a national baseline of hypoglycaemia and confirm earlier reports (80) of daily hypoglycaemia patterns at a group level. Finally, Park et al. (83) recently reported preliminary data from the GRACE trial, establishing the extent of glucose variability in children with adrenal insufficiency compared to healthy controls.

### 7.2 CGM as a behaviour change tool

CGM is still in its infancy as a technology and new ways are being explored to derive positive impact for patients’ health. Traditional usage has focused on high frequency glucose data to allow patients to adjust insulin doses and to predict upcoming excursions from euglycaemia. As discussed above, CGM has been adopted by the computer science community with a focus on the development of glucose forecasting algorithms (64, 84) to improve the accuracy with which these excursions are predicted.

However, a new direction for CGM use is now being investigated, CGM as a behaviour change tool. In their review, Ehrhardt and Zaghal (85) conclude that “Rather than being used as a “reactionary device” for hypoglycaemia prevention and glycaemic management, CGM should be assessed for its use as a prevention tool. Its potential role as an adjunct to lifestyle changes [ … ] calls for further evaluation”. In a survey of 40 people living with diabetes (86), 90% commented that CGM contributed to a healthier lifestyle, with 87% modifying food choices and 47% increasing physical activity based on CGM. Recent publications have also suggested that CGM could act as a behaviour modification tool for those with obesity (87).

Combining pattern recognition with behaviour change has the potential to significantly improve self-management behaviours (88). Worth et al. used CGM to identify individual patterns in weekly hypoglycaemia risk of patients with CHI (81). The same group developed interpretative algorithms to facilitate patient understanding of patterns and provided suggestions for reflection designed to modify parental behaviours (35). The resulting change in fingerprick and self-management behaviours led to a reduction in real world hypoglycaemia of 25% (35, 81), demonstrating the potential power of using CGM as a tool to identify and modify the behavioural determinants of hypoglycaemia. Due to the focus on weekly patterns and behavioural determinants of hypoglycaemia, this approach is less subject to problems with poor point accuracy and patient dissatisfaction with alarms, suggesting a novel and sustainable path to CGM application.

### 7.3 CGM to diagnose and inform management

While children with rare hypoglycaemia disorders do not have exogenous insulin to adjust based on CGM readings, there are many other diagnostic and management decisions that can be made upon the basis of CGM outputs. Work evaluating the CGM profiles of healthy subjects (89, 90) provides more data with which researchers can compare results from disease cohorts and evaluate glycaemic control in context. Rossi et al. (30) have shown this with their own assessment of healthy subjects in comparison to those with GSDIa. Separately, Rossi et al. (91) propose the use of CGM in a hybrid approach to determine fasting tolerance in children with GSDs rather than the traditional “controlled fast” with multiple fingerprick tests. They go on to highlight the efficacy of CGM to determine incidence of nocturnal hypoglycaemia as well as the impact of diet and medications on glycaemic profiles. Peeks et al. (42) support this approach and have documented their use of CGM to monitor the impact of nocturnal dietary interventions, changes in starch loads, and treatment with empagliflozin for patients with hepatic GSDs. In the case of treatment with empagliflozin, the authors highlight the utility of CGM to detect the potential hypoglycaemia resulting from medication-induced glycosuria (42). Logel et al. (92) similarly used intermittent CGM to initiate and then titrate doses of diazoxide in a patient with Glut1 deficiency who had failed ketogenic diet; without the high granularity data of CGM it was felt that diazoxide would have been administered at incorrect doses, risking the loss of efficacy seen in other cases treated without CGM.

### 7.4 CGM as an outcome marker in clinical trials

In recent years CGM has become popular as an outcome in clinical trials to determine efficacy of interventions to reduce hypoglycaemia. The high granularity data generated by CGM reduces the chance of type II errors in clinical trials and allows investigators better insight into glycaemic changes secondary to therapeutics.

CGM has recently been used as an outcome measure for: hypoglycaemia after paediatric cardiac surgery (93); treatment of CHI with Dasiglucagon (94); treatment of CHI with RZ358 (95); treatment of GSDIa with AAV8 gene transfer (96) and is planned for more upcoming therapeutic trials in rare hypoglycaemia disorders. An essential component of using CGM as an outcome measure is understanding the baseline data for each disease and population (42). This requires quantification of as many patients as possible (79); Rossi et al. (30) recently provided the first publication of CGM metrics for patients with GSD1a, as did Worth et al. (80, 82) for patients with CHI, essential datasets for those utilising baseline characteristics when designing future therapeutic trials using CGM for primary or secondary outcomes.

## 8 Conclusion

There has been considerable progress in the development of the relatively new technology of CGM. However, in childhood hypoglycaemia disorders many historical problems remain. CGM continues to be insufficiently accurate, somewhat burdensome for patients and their families, costly, and lacking in evidence for its ability to reduce hypoglycaemia when provided to families without support. However, there is scope for optimism. Devices continue to miniaturise, improve in accuracy and reduce patient burden. Research and clinical teams are working around suboptimal point accuracy and lack of patient educational resources to develop novel ways of utilising this technology. CGM is being used for diagnostics, monitoring changes in management, establishment of baseline characteristics, modifying behaviour, and ultimately to reduce hypoglycaemia when used retrospectively and combined with interpretative algorithms or clinical expertise. Use in neonatal medicine is becoming established, with good evidence for a reduction and early recognition in neonatal hypoglycaemia.

A lack of guidelines for the use of CGM in hypoglycaemia disorders has restricted progress but given rapid technological advances, it is predicted to play a larger role in all forms of childhood hypoglycaemia disorders. The challenge is to adapt CGM technology to clinical application with research designed to bring CGM innovations for patient benefit.

## Author contributions

CW researched and wrote the first draft of the manuscript other than Section 5.2 which was written by LH. All authors contributed to the article and approved the submitted version.
